# Exposure to Magnetic Field Non-Ionizing Radiation and the Risk of Miscarriage: A Prospective Cohort Study

**DOI:** 10.1038/s41598-017-16623-8

**Published:** 2017-12-13

**Authors:** De-Kun Li, Hong Chen, Jeannette R. Ferber, Roxana Odouli, Charles Quesenberry

**Affiliations:** Division of Research, Kaiser Foundation Research Institute, Kaiser Permanente, Oakland, CA USA

## Abstract

Magnetic field (MF) non-ionizing radiation is widespread and everyone is exposed to some degree. This prospective cohort study of 913 pregnant women examined the association between high MF exposure and miscarriage risk. Cox (proportional hazards) regression was used to examine the association. After controlling for multiple other factors, women who were exposed to higher MF levels had 2.72 times the risk of miscarriage (hazard ratio = 2.72, 95% CI: 1.42–5.19) than those with lower MF exposure. The increased risk of miscarriage associated with high MF was consistently observed regardless of the sources of high MF. The association was much stronger if MF was measured on a typical day of participants’ pregnancies. The finding also demonstrated that accurate measurement of MF exposure is vital for examining MF health effects. This study provides fresh evidence, directly from a human population, that MF non-ionizing radiation could have adverse biological impacts on human health.

## Introduction

Magnetic field (MF) non-ionizing radiation is a ubiquitous environmental exposure and a serious looming public health challenge. MFs are emitted from both traditional sources that generate low frequency MFs (e.g., power lines, appliances, transformers, etc.) and from emerging sources that generate higher frequency MFs (e.g., wireless networks, smart meter networks, cell towers, wireless devices such as cell phones, etc.). Humans are now widely exposed to MF with ever-increasing intensity, due to the proliferation of MF-generating apparatuses.

The steep increase in MF exposure has renewed concerns about the potential health effects of this invisible, man-made environmental exposure. A recent NIEHS multi-year project conducted by the National Toxicology Program (NTP) has revealed an increased risk of cancer associated with MF non-ionizing radiation exposure^1,2^. More specifically, the NTP study found that the cancer risk due to MF exposure observed in their experimental animals matched the cancer cell types that had been reported in previous epidemiologic studies in human populations^1^. This finding has made it more difficult to continue to dismiss possible biological effects of MF exposure. Such outright dismissal could be especially troublesome given the high prevalence of human exposure (with almost everyone being exposed to MF non-ionizing radiation to some degree). This includes vulnerable populations such as pregnant women and young children. The International Agency for Research on Cancer (IARC) has classified MF as a possible carcinogen^3,4^.

Miscarriage is one of the potential adverse health outcomes that are sensitive to MF exposure and also an endpoint that the WHO has recommended to be further studied in the context of MF health effects^5^. Over the years, a few observational studies in human populations have suggested a possible link between MF exposure during pregnancy and an increased risk of miscarriage^6–11^ including two studies published in 2002 that increased the public awareness of such an association^12,13^. In addition, one study examined human embryonic tissues to assess the association between EMF exposure and embryonic growth, and observed an increased risk of impaired embryonic bud growth and apoptosis associated with exposure to higher MF level^14^, providing some direct evidence of adverse biological impact of EMF exposure on embryonic development.

Nevertheless, the association between MF exposure and risk of miscarriage remains largely unknown and overlooked. We conducted this prospective cohort study among a large population of pregnant women to further examine whether exposure to MF non-ionizing radiation during pregnancy increases the risk of miscarriage.

## Materials and Methods

This prospective cohort study was approved by the Kaiser Permanente Northern California (KPNC) Institutional Review Board and conducted among KPNC’s pregnant members in the San Francisco Bay Area, all of whom provided informed consent. The study was performed in accordance with all relevant guidelines and regulations. KPNC is an integrated health care delivery system whose members comprise 28–30% of the population in the catchment area and have consistently been shown to be representative of the underlying population^15,16^.

### Study population

All pregnant women, aged 18 years or older, and residing in the participating Bay Area counties, were identified through the KPNC electronic medical record (EMR) laboratory database based on positive pregnancy tests. At KPNC, all women suspected to be pregnant were routinely asked to have a pregnancy test done at a KPNC facility. Flyers informing women about the study were posted at the participating facilities and given to women at the time of their pregnancy test. Given that miscarriage can occur very early in pregnancy, recruiting pregnant women as early as possible in their pregnancy was crucial to ensuring as complete ascertainment of miscarriage as possible. Our identification of pregnant women through positive pregnancy lab tests ensured early recruitment. To determine whether a woman’s recurrent miscarriage(s), an indication of higher susceptibility to miscarriage, increases her vulnerability to MF exposure, we oversampled women with two or more prior miscarriages. The pregnant women identified were contacted by a trained recruiter/interviewer to determine their eligibility and willingness to participate in the study. Those who indicated their intention to carry the pregnancy to term and whose gestational age at identification was less than 10 completed weeks (still at risk for miscarriage) were invited to participate in the study. Among 1,627 eligible pregnant women, 1,054 agreed to participate in the study.

### Measuring magnetic field exposure during pregnancy

All participating pregnant women were asked to carry an EMDEX Lite meter (Enertech Consultants Inc.) for 24 hours during pregnancy. The EMDEX Lite  meter is specifically designed to measure MF, which is measured in milligauss (mG).

To ensure better representation of MF exposure during pregnancy and to apply the knowledge gained from the previous study^12^, we designed the MF measurement to be conducted on a typical day (a day reflecting participants’ typical pattern of work and leisure activities during pregnancy). In the event that a participant’s daily activities might have been altered from what was originally planned, we also verified with the participants, at the end of the measurement period, whether the measurement day was indeed a typical day of their pregnancy. If not, the measurement day was classified as non-typical.

The EMDEX Lite meter was used to measure MF exposure levels by participating pregnant women from all emitting sources. Participants were also asked to keep a diary during the 24-hour measurement period to allow the researchers to (1) identify locations of daily activities (at home, at home in bed, in transit, at work, and other), (2) verify if activities were reflective of a typical day, and (3) examine if locations and activities were associated with high MF exposure.

MF data together with participants’ diary of activities on the measurement day were examined for quality control, including consistency and potential errors. We excluded 31 subjects who failed to carry the meter as instructed. We also excluded 107 subjects who had incomplete (<90% of their 24-hour measurements) MF measurement data. Those exclusions were made without knowledge of subjects’ pregnancy outcomes.

Previous studies have found that the highest MF levels that pregnant women encounter are the most relevant to miscarriage risk^12,13^, indicating a possible threshold effect at a given MF level above which developmental embryos may cease to be viable. Thus, this study focused on high levels of MF exposure. We used the 99^th^ percentile of MF measurements during the 24-hour period to classify exposure level, balancing between the need to examine as high of MF level as possible and, at the same time, avoid using less stable indices (e.g., maximum exposure level).

To more accurately reflect participants’ true MF exposure during pregnancy, we made significant efforts to separate those participants whose measurements were conducted during a typical day of their pregnancy from those whose measurements were not conducted on a typical day. Measurements obtained on a *typical day* are likely more representative of MF exposure during pregnancy while measurements obtained on a *non-typical* day are more subject to misrepresentation of the true MF exposure level during pregnancy, resulting in misclassifying participants into incorrect MF exposure categories. Such misclassification usually reduces scientists’ ability to detect an underlying association. As demonstrated in a previous study, measurements conducted on a typical day showed a stronger association between MF exposure and miscarriage risk, while measurements conducted on a non-typical day showed virtually no association due to incorrectly classifying participants into MF exposure categories^12^.

### Measurement of miscarriage

Using KPNC EMR data, we were able to identify participants’ pregnancy immediately after a positive pregnancy test, thereby starting follow-up at an earlier gestational age than the first prenatal visit, the earliest time at which most other studies have been able to identify pregnant women. This early follow-up allowed us to ascertain early miscarriages that most other studies would have missed, making it an important strength of this study.

All participants were followed for their pregnancy outcomes from the time of their positive pregnancy test to the end of their pregnancy. In the case of miscarriage, this is, by definition, before 20 completed weeks of gestation. We ascertained pregnancy outcomes through the KPNC EMR databases. For participants whose outcomes were not available in the EMR, we contacted them directly. We were able to identify pregnancy outcomes for all participants except one who had moved out of the area, thus she was excluded from further analysis.

### In-person interview

An *in-person* interview was conducted with all participants to ascertain extensive information on potential confounders, including pregnancy history and risk factors for miscarriage. Previous studies have shown that MF exposure level is seldom related to common socio-demographic characteristics and risk factors^12,17,18^; thus, the number of potential confounders in this study was small. Nevertheless, we still collected many factors for examination to ensure thorough control of confounders. Two participants were not able to complete the interview, thus they were excluded from the analyses.

The prospective study design also ensured that the *in-person* interview was blinded to MF exposure for both interviewers and participants, since the EMF measurement was conducted after the interview. This study design enhances the quality of the study findings.

### Statistical analysis

We used the Cox Proportional Hazards regression model, with accommodation for left truncation, to examine the association between MF exposure level and miscarriage. Hazard ratios with 95% confidence intervals were used to determine the magnitude and significance of associations. Left truncation arises when study participants enter observation at a point in time (i.e. gestational age at cohort entry) after the time of origin, conception. Participants were followed until either (a) miscarriage, (b) end of pregnancy due to other outcomes (e.g., ectopic pregnancy), at which point they were censored or (c) 20 weeks of gestation, for participants who remained pregnant at that time.

We examined confounders using the change-in-estimate criterion, including the confounder if the miscarriage hazard ratio (HR) for MF changed by 10% or more. While most factors examined were not confounders due to a lack of association with MF exposure, we nevertheless included in the model commonly known risk factors for miscarriage and socio-demographic characteristics.

Given the previous finding that the strength of association between MF and miscarriage varied by whether the MF measurements were taken on a typical or non-typical day^12^, we first conducted analyses separately by day type. The previous finding was confirmed in the current study, and we therefore conducted the remaining analyses only among those whose MF exposure was measured on *a typical day* of their pregnancy.

Since we oversampled those with multiple prior miscarriages, we first stratified analysis by those with and without multiple prior miscarriages to determine if the MF association with miscarriage risk differed between these two groups. Once it was determined that the observed associations were largely similar, we included all participants in the analyses and adjusted for prior miscarriage in all the models.

A total of 913 subjects with valid MF measurements and pregnancy outcomes were included in the final analysis.

Statistical analyses were conducted using SAS 9.3.

## Results

Table 1 presents the description and characteristics of participants based on their MF exposure levels (high vs. low). The low MF exposure group consisted of women whose 99^th^ percentile of MF exposure levels was in the lowest quartile (<2.5 mG), while those in the higher three quartiles were classified in the high MF exposure group. There were no noticeable associations or consistent patterns between MF exposure level and most of the factors examined, including risk factors for miscarriage (Table 1).

**Table 1:** Characteristics of the Study Population by Daily Magnetic Field Exposure Level (Lowest or Higher Quartiles of MF 99^th^ Percentile).

Characteristic	Total N (N = 913)^a^	99^th^ Percentile MF Level			
		Lowest quartile (N = 219)		Higher quartiles (N = 694)	
		N	%	N	%
**Maternal age**					
<30	296	61	*27*.*9%*	235	*33*.*9%*
30–34	288	71	*32*.*4%*	217	*31*.*3%*
≥35	329	87	*39*.*7%*	242	*34*.*9%*
**Race**					
White	326	91	*41*.*7%*	235	*34*.*0%*
Black	90	16	*7*.*3%*	74	*10*.*7%*
Hispanic	226	51	*23*.*4%*	175	*25*.*3%*
Asian /Pacific Islander	202	44	*20*.*2%*	158	*22*.*8%*
Other	66	16	*7*.*3%*	50	*7*.*2%*
**Education**					
<High school	42	5	*2*.*3%*	37	*5*.*4%*
High school or GED	142	32	*14*.*7%*	110	*15*.*9%*
Trade/Technical school	46	5	*2*.*3%*	41	*5*.*9%*
College degree	495	128	*58*.*7%*	367	*53*.*1%*
Graduate school	184	48	*22*.*0%*	136	*19*.*7%*
**Marital Status**					
Single	72	12	*5*.*5%*	60	*8*.*7%*
Partnered	147	31	*14*.*2%*	116	*16*.*8%*
Married	690	175	*80*.*3%*	515	*74*.*5%*
**Worked in last year**					
No	183	47	*21*.*6%*	136	*19*.*7%*
Yes	727	171	*78*.*4%*	556	*80*.*3%*
**Smoked since LMP**					
No	807	196	*91*.*2%*	611	*89*.*3%*
Yes	92	19	*8*.*8%*	73	*10*.*7%*
**Coffee intake since LMP**					
0 cup/day	637	142	*64*.*8%*	495	*71*.*3%*
0–1 cup/day	201	52	*23*.*7%*	149	*21*.*5%*
>1 cups/day	75	25	*11*.*4%*	50	*7*.*2%*
**Alcohol use since LMP**					
No	514	127	*58*.*3%*	387	*55*.*8%*
Yes	397	91	*41*.*7%*	306	*44*.*2%*
**Number of previous pregnancies**					
0	94	21	*9*.*6%*	73	*10*.*5%*
1	103	18	*8*.*2%*	85	*12*.*2%*
2	140	36	*16*.*4%*	104	*15*.*0%*
≥3	576	144	*65*.*8%*	432	*62*.*2%*
**Number of previous miscarriages**					
0	276	60	*27*.*4%*	216	*31*.*1%*
1	79	21	*9*.*6%*	58	*8*.*4%*
2	403	101	*46*.*1%*	302	*43*.*5%*
≥3	155	37	*16*.*9%*	118	*17*.*0%*
**History of subfertility**					
No	633	147	*67*.*1%*	486	*70*.*0%*
Yes	280	72	*32*.*9%*	208	*30*.*0%*
**Vaginal bleeding since LMP**					
No	670	165	*75*.*7%*	505	*72*.*9%*
Yes	241	53	*24*.*3%*	188	*27*.*1%*
**Urinary tract infection since LMP**					
No	860	211	*96*.*8%*	649	*93*.*9%*
Yes	49	7	*3*.*2%*	42	*6*.*1%*
**Fever since LMP**					
No	851	198	*92*.*1%*	653	*94*.*8%*
Yes	53	17	*7*.*9%*	36	*5*.*2%*
**Carry loads** (**>10 pounds**) **since LMP**					
No	416	92	*42*.*2%*	324	*46*.*8%*
Yes	494	126	*57*.*8%*	368	*53*.*2%*
**Used Jacuzzi/hot tub/steam room/sauna since LMP**					
No	807	200	*91*.*7%*	607	*87*.*7%*
Yes	103	18	*8*.*3%*	85	*12*.*3%*
**Exposure to solvents or degreasers since LMP**					
No	609	148	*68*.*5%*	461	*67*.*7%*
Yes	288	68	*31*.*5%*	220	*32*.*3%*
**Vitamin use since LMP**					
No	91	16	*7*.*3%*	75	*10*.*8%*
Yes	820	202	*92*.*7%*	618	*89*.*2%*
**Gestational age at study entry**					
0–48 days	763	173	*79*.*0%*	590	*85*.*0%*
49–69 days	135	41	*18*.*7%*	94	*13*.*5%*
≥70 days	15	5	*2*.*3%*	10	*1*.*4%*

Abbreviation: LMP, Last menstrual period.

^a^The numbers in each individual category may not sum to the total number because of missing data.

After adjustment for maternal age, race, education, smoking during pregnancy, and prior miscarriage, overall, pregnant women who had higher MF exposure during pregnancy (higher 3 quartiles) had a 48% greater risk of miscarriage than women who had lower MF exposure (in the lowest quartile): adjusted HR = 1.48, 95% confidence interval (CI): 1.03–2.14 (Table 2). Notably, consistent with the finding in a prior study^12^, the observed association was much stronger among participants whose MF exposure was measured on a typical day of the pregnancy (aHR = 2.72, 1.42–5.19). In contrast, there was no observed association among those whose MF was measured on a non-typical day (Table 2). Thus, the following analyses were restricted to those whose MF was measured on a typical day of their pregnancy.

**Table 2:** Exposure to High Magnetic Fields (MFs) During Pregnancy and the Risk of Miscarriage.

99^th^ Percentile MF Level	Total N	Miscarriage N (*%*)	cHR (95% CI)	aHR^a^ (95% CI)
**Among all participants**				
Lowest quartile	219	36 (*16*.*4%*)	Ref	Ref
Higher quartiles	694	164 (*23*.*6%*)	1.43 (1.00–2.06)	1.48 (1.03–2.14)
**MF measured on** ***typical days***				
Lowest quartile	106	11 (*10*.*4%*)	Ref	Ref
Higher quartiles	347	84 (*24*.*2%*)	2.46 (1.31–4.62)	**2**.**72** (1.42–5.19)
**MF measured on non-typical days**				
Lowest quartile	113	25 (*22*.*1%*)	Ref	Ref
Higher quartiles	347	80 (*23*.*1%*)	1.02 (0.65–1.62)	1.08 (0.67–1.73)

cHR: crude (unadjusted) hazard ratio; aHR: adjusted hazard ratio. 95% CI: 95% Confidence interval.

^a^Adjusted for maternal age at interview, race, education, smoking since LMP and prior miscarriage.

Further adjustment for the following variables did not change the results: maternal nausea/vomiting, maternal income, marital status, alcohol use, caffeine intake, maternal fever, vaginal bleeding, urinary tract infection, carrying loads > 10lbs, exposure to solvents or degreasers, vitamin intake and Jacuzzi/hot tub/steam room/sauna use during pregnancy.

Next, we examined the association separately among women with and without multiple prior miscarriages (≥2). Table 3 showed that the association was largely similar between these two groups, with the association being slightly stronger among women without multiple prior miscarriages.

**Table 3:** Exposure to High Magnetic Fields (MFs) During Pregnancy and the Risk of Miscarriage, Stratified by Number of Prior Miscarriages, *MF Measured on Typical Days Only*.

99^th^ Percentile MF Level	Total N	Miscarriage N (*%*)	cHR (95% CI)	aHR^a^ (95% CI)
**≤1 prior miscarriages**				
Lowest quartile	39	3 (*7*.*7%*)	Ref	Ref
Higher quartiles	143	27 (*18*.*9%*)	**2**.**69** (0.82–8.87)	**3**.**76** (1.07–13.18)
**≥2 prior miscarriages**				
Lowest quartile	67	8 (*11*.*9%*)	Ref	Ref
Higher quartiles	204	57 (*27*.*9%*)	**2**.**43** (1.16–5.11)	**2**.**56** (1.19–5.50)

cHR: crude (unadjusted) hazard ratio; aHR: adjusted hazard ratio. 95% CI: 95% Confidence interval.

^a^Adjusted for maternal age at interview, race, education, smoking since LMP, and gravidity.

Further adjustment for the following variables did not change the results: maternal nausea/vomiting, maternal income, marital status, alcohol use, caffeine intake, maternal fever, vaginal bleeding, urinary tract infection, carrying loads > 10lbs, exposure to solvents or degreasers, vitamin intake and Jacuzzi/hot tub/steam room/sauna use during pregnancy.

Table 4 shows the possible dose-response relationship by examining the association for each quartile using the lowest quartile (2.5 mG) as the reference group. While all higher quartiles showed an increased risk of miscarriage compared to the lowest MF exposure group, there was no dose-response relationship observed. These results are similar to those of a prior study^12^.

**Table 4:** Exposure to High Magnetic Fields (MFs) During Pregnancy and the Risk of Miscarriage – Assessing Dose-Response, *MF Measured on Typical Days Only*.

99^th^ Percentile MF Level	Total N	Miscarriage N (*%*)	cHR (95% CI)	aHR^a^ (95% CI)
1^st^ quartile (<2.5 mG)	106	11 (*10*.*4%*)	Ref	Ref
2^nd^ quartile (2.5–3.6 mG)	116	32 (*27*.*6%*)	2.87 (1.45–5.70)	3.29 (1.59–6.79)
3^rd^ quartile (3.7–6.2 mG)	119	31 (*26*.*1%*)	2.70 (1.36–5.39)	3.01 (1.48–6.12)
4^th^ quartile (≥6.3 mG)	112	21 (*18.8%*)	1.83 (0.88–3.79)	2.02 (0.95–4.28)

cHR: crude (unadjusted) hazard ratio; aHR: adjusted hazard ratio. 95% CI: 95% Confidence interval.

^a^Adjusted for maternal age at interview, race, education, smoking since LMP, and prior miscarriage.

Further adjustment for the following variables did not change the results: maternal nausea/vomiting, maternal income, marital status, alcohol use, caffeine intake, maternal fever, vaginal bleeding, urinary tract infection, carrying loads > 10lbs, exposure to solvents or degreasers, vitamin intake and Jacuzzi/hot tub/steam room/sauna use during pregnancy.

The above-observed association was consistent regardless of the source of the MF. Although we did not have information on the exact sources from which MF was generated, based on participants’ diary, we were able to examine whether MF exposure was from any of the following location categories: at home, at home in bed, at work, in transit, or from other sources. The association was observed consistently, regardless of the location. In addition to the adjusted variables mentioned above, further adjustment for nausea and vomiting as well as the following variables did not change the results in Tables 2–4: maternal income, marital status, maternal nausea/vomiting, alcohol use, caffeine intake, maternal fever, vaginal bleeding, urinary tract infection, carrying loads > 10 pounds, exposure to solvents or degreasers, vitamin intake, and Jacuzzi/hot tub/steam room/sauna use during pregnancy.

## Discussion

After initial reports that provided evidence of an increased risk of miscarriage associated with high MF exposure during pregnancy^12,13^, the current NIEHS-funded study provides additional evidence that exposure to high MF levels in pregnancy is associated with increased risk of miscarriage. This finding is also supported by four other studies published during the past 15 years that examined the relationship between high MF exposure and the risk of miscarriage^8–11,19^. Two of those studies measured EMF both inside, and in the surrounding areas, of the residence of participating pregnant women, and observed a higher risk of miscarriage associated with higher EMF exposure levels^8,9^. Two other studies examined the impact of EMF emitted from cell phones and wireless networks, and observed that more frequent cell phone use and close proximity to wireless base stations were both associated with an increased risk of miscarriage^10,11^. Although none of these studies conducted any personal MF measurements to capture actual MF exposure from all sources, as the current study has done, all four studies reported an increased risk of miscarriage associated with high MF exposure.

One of the most challenging aspects of assessing the health impact of MF exposure is the ability to measure MF exposure accurately as well as in the relevant etiological period. Prospectively measuring MF exposure in the etiologically relevant timeframe is essential and preferable to retrospective measurements. It is especially problematic to ascertain MF exposure long after the relevant window of exposure has passed. While logistically challenging, a prospective study design with a device that captures actual MF levels from all emitting sources in an etiologically relevant period will notably improve the accuracy of MF exposure assessment in epidemiological studies in a human population. In addition, as both this study and a previous study^12^ demonstrated, even with a prospective design, if measurements were not conducted on a typical day to reflect true MF exposure during pregnancy, such study design could still fail to detect any MF health risk due to misclassification of MF exposure (see Table 2). Therefore, to ensure accurate exposure assessment, MF measurements need to be conducted prospectively during an etiologically relevant window *and* to reflect a participant’s typical MF exposure patterns. The determination of whether the activity pattern was typical needs to be verified after measurement is complete since planned activities can change during the measurement day. It is clear that, if MF exposure is measured subjectively (e.g., interview based on participants’ recall) or based on surrogate measures (e.g., wire codes, distance from power lines, job matrix, spot measurement at home, etc.), it would be very difficult for such studies to detect any MF health effect in epidemiological studies due to gross inaccuracies in measuring actual MF exposure levels. By definition, inaccurate MF measures lead to misclassification of MF exposure, which generally result in null findings. Unfortunately, the vast majority of epidemiological studies on MF health effects in the literature so far have been based on subjective and unreliable MF measurements. Thus, it is not surprising that many of the past studies failed to detect MF health effects. In addition, the focus on studying MF effects on cancer has exacerbated the problem, since the development of cancer usually has a long latency period between exposure and outcome that could span several decades. This has made accurately measure MF exposure in the etiologically relevant period (decades before the diagnosis of cancer) almost impossible. Those “null findings” have left a false impression of the “safety” of MF exposure.

The strength of this current study is that, in addition to using an objective measuring device (EMDEX Lite meter), we examined an outcome (miscarriage) with a short latency period (days or weeks rather than years or decades as in the case of cancers or autoimmune diseases). Thus, we were able to measure MF exposure prospectively in the relevant time period (during pregnancy). Furthermore, at the end of the measurement day, we ascertained whether activity patterns on that day reflected a typical day, which allowed us to identify participants with MF exposure measurements that more accurately reflected MF exposure during their pregnancies.

In this study, we found an almost three-fold increased risk of miscarriage if a pregnant woman was exposed to higher MF levels compared to women with lower MF exposure. The association was independent of any specific MF exposure sources or locations, thus removing the concern that other factors connected to the sources of the exposure might account for the observed associations. While nausea and vomiting were hypothesized to be potential confounders, adjustment for both nausea and vomiting did not change the results in this study or in a previous study^20^. Although we did not observe a dose-response relationship for MF exposure above 2.5 mG, this could be due to a threshold effect of MF exposure in which MF levels at or above 2.5 mG could lead to fetal demise, thus examining further higher levels of MF exposure were not able to confer additional risk.

Given the ubiquitous nature of exposure to this non-ionizing radiation, a small increased risk due to MF exposure could lead to unacceptable health consequences to pregnant women. Although the number of epidemiological studies examining the adverse impact of MF exposure in humans remains limited, the findings of this study should bring attention to this potentially important environmental hazard to pregnant women, at least in the context of miscarriage risk, and stimulate much needed additional research.

## References

[1] Wyde, M. *et al*. *Report of Partial findings from the National Toxicology Program Carcinogenesis Studies of Cell Phone Radiofrequency Radiation in Hsd: Sprague Dawley® SD rats* (*Whole Body Exposure*), http://biorxiv.org/content/early/2016/06/23/055699 (2016).

[2] National Toxicology Program. *Media**Telebriefing**:**NTP**Cell Phone Radiofrequency Radiation Study: Partial Release of Findings*, http://www.niehs.nih.gov/news/newsroom/releases/2016/may27/ (2016).

[3] Baan R (2011). Carcinogenicity of radiofrequency electromagnetic fields. The Lancet. Oncology.

[4] International Agency for Research on Cancer Working Group on the Evaluation of Carcinogenic Risks to Humans. *Non-Ionizing Radiation*, *Part 2: Radiofrequency Electromagnetic Fields*. Vol. 102 (World Health Organization, 2013). PMC4780878 24772662

[5] World Health Organization. 2007 WHO Research Agenda for Extremely Low Frequency Fields. (World Health Organization, Geneva, Switzerland, 2007).

[6] Lindbohm ML (1992). Magnetic fields of video display terminals and spontaneous abortion. Am.J.Epidemiol..

[7] Juutilainen J, Matilainen P, Saarikoski S, Laara E, Suonio S (1993). Early pregnancy loss and exposure to 50-Hz magnetic fields. Bioelectromagnetics.

[8] Wang Q (2013). Residential exposure to 50 Hz magnetic fields and the association with miscarriage risk: a 2-year prospective cohort study. PLoS One.

[9] Shamsi MF, Ziaei S, Firoozabadi M, Kazemnejad A (2013). Exposure to Extremely Low Frequency Electromagnetic Fields during Pregnancy and the Risk of Spontaneous Abortion: A Case-Control Study. J Res Health Sci.

[10] Zhou LY (2015). Epidemiological investigation of risk factors of the pregnant women with early spontaneous abortion in Beijing. Chin J Integr Med.

[11] Mahmoudabadi FS, Ziaei S, Firoozabadi M, Kazemnejad A (2015). Use of mobile phone during pregnancy and the risk of spontaneous abortion. J Environ Health Sci Eng.

[12] Li DK (2002). A population-based prospective cohort study of personal exposure to magnetic fields during pregnancy and the risk of miscarriage. Epidemiology.

[13] Lee GM, Neutra RR, Hristova L, Yost M, Hiatt RA (2002). A nested case-control study of residential and personal magnetic field measures and miscarriages. Epidemiology.

[14] Su XJ (2014). Correlation between Exposure to Magnetic Fields and Embryonic Development in the First Trimester. PLoS One..

[15] Gordon, N. P. A Comparison of Sociodemographic and Health Characteristics of the Kaiser Permanente Northern California Membership Derived from Two Data Sources: The 2008 Member Health Survey and the 2007 California Health Interview Survey., (Kaiser Permanente Division of Research, Oakland, CA, 2012).

[16] Gordon, N. P. Similarity of the Adult Kaiser Permanente Membership in Northern California to the Insured and General Population in Northern California: Statistics from the 2011–12 California Health Interview Survey. (Kaiser Permanente Division of Research, Oakland, CA, 2015).

[17] Li, D. K., Chen, H. & Odouli, R. Maternal Exposure to Magnetic Fields During Pregnancy in Relation to the Risk of Asthma in Offspring. *Arch*.*Pediatr*.*Adolesc*.*Med*. (2011). 10.1001/archpediatrics.2011.135 21810627

[18] Li DK, Ferber JR, Odouli R, Quesenberry CP (2012). A prospective study of in-utero exposure to magnetic fields and the risk of childhood obesity. Sci.Rep..

[19] Shah, S. G. & Farrow, A. Systematic Literature Review of Adverse Reproductive Outcomes Associated with Physiotherapists’ Occupational Exposures to Non-ionising Radiation. *J Occup*.*Health* (2014). 10.1539/joh.13-0196-ra 25069893

[20] Li DK, Neutra RR (2002). Magnetic fields and miscarriage. Epidemiology.

