# Editorial Expression of Concern: Exposure to Magnetic Field Non-Ionizing Radiation and the Risk of Miscarriage: A Prospective Cohort Study

**DOI:** 10.1038/s41598-021-85479-w

**Published:** 2021-03-10

**Authors:** De-Kun Li, Hong Chen, Jeannette R. Ferber, Roxana Odouli, Charles Quesenberry

**Affiliations:** Division of Research, Kaiser Foundation Research Institute, Kaiser Permanente, Oakland, CA USA

Editorial Expression of Concern to: *Scientific Reports* 10.1038/s41598-017-16623-8, published online 13 December 2017

The Editors are issuing an Editorial Expression of Concern for this Article.

The Editors have recently been made aware by the Authors that the data underlying this study cannot be shared. Unfortunately, the Authors did not obtain participant consent for data sharing, and it is not possible for them to do this retrospectively. Therefore the data are not available for request by readers wishing to repeat the analyses presented in this work.

All Authors agree with this Editorial Expression of Concern and its wording.

